# Perception of medical professionalism among the surgical community in the University of Nairobi: a mixed method study

**DOI:** 10.1186/s12909-016-0622-4

**Published:** 2016-04-01

**Authors:** Daniel Kinyuru Ojuka, Joyce M. Olenja, Nimrod J. Mwango’mbe, Eunbae B. Yang, Jana B. Macleod

**Affiliations:** Department of Surgery, College of Health Sciences, University of Nairobi, P. O. Box 19676–00202, Nairobi, Kenya; School of Public Health, College of Health Sciences, University of Nairobi, Nairobi, Kenya; Department of Medical Education, Yonsei University, Seoul, South Korea; Department of Surgery, School of Medicine, Kenyatta University, Nairobi, Kenya

## Abstract

**Background:**

Professionalism defines the relationship between colleagues, patients and the society as a whole. Furthermore, being a social construct, professionalism is sophisticated to be regarded simply as a single concept across different cultural contexts. This study sought to explore how professionalism is conceptualized by the clinicians, students and patients in a teaching hospital in Kenya.

**Methods:**

A sequential mixed methods study was conducted among clinicians, students and patients at Kenyatta National Hospital on the surgical wards from March 1^st^-December 31^st^, 2014. The first phase of the study involved focus group discussions (FGDs) of between 10–12 persons and individual in-depth interviews of senior faculty and patients. Grounded theory method was used for collecting perceptions of participants on professionalism. These views were then coded using Atlas 5.2, allowing the development of a questionnaire that provided the survey tool for the second phase of the study. For the questionnaire, response options utilized a 4-point Likert scale with a range from “strongly agree” to “strongly disagree”. Factor analysis was used to analyse the responses to the survey. Internal reliability was determined by Cronbach’s α.

**Results:**

Sixteen FGDs and 18 in-depth interviews were held with 204 clinicians, students and patients. A further 188 participants completed the questionnaire. Respect was the most frequently mentioned or picked component of professionalism during the interview and survey respectively, with 74.5 % of participants reporting “strongly agree”. Factor analysis showed that 3 factors accounted for the majority of the variance in the items analysed; respect in practice, excellence in service and concern for the patient. The Cronbach’s α for this analysis was 0.927.

**Conclusion:**

The study cohort predominantly conceptualizes professionalism as relating to respect between colleagues and toward patients. Respect, being a cultural norm, should form part of the core curriculum of professionalism in order to be relevant for the Kenyan context.

## Background

Health care worker professionalism has become a subject of great concern to the public, those who teach medicine and practicing physicians themselves [1]. In Kenya, various newspaper articles have highlighted medical cases that have demonstrated lack of professionalism. A recent article appearing in the *Standard Newspaper* alleged that a doctor left a patient on the operating room table with an un-sutured surgical wound and went to the pub to drink alcohol. A similar article in the *Daily Nation* alleged that a doctor was paid by a member of parliament to forcefully perform an HIV test on a young woman with whom the MP wanted to have sexual relations [2, 3]. Such stories, though not substantiated, demonstrate a severe lack of professionalism, and reflects the depth of the problem and its wide variation in occurrence. Ultimately, such unprofessional and unethical behaviour damages public trust in physicians and in the medical services as a whole.

Historically, physicians have been well respected by their communities and viewed as professional healers with high standards of behaviour. Although the role of the healer has not changed, the understanding of professionalism has been impacted by societal pressures and professional needs. There have been many changes in the practise of medicine over the recent years which have impacted the professional attitudes and behaviours of health care workers. These changes includes; technological advances leading to reduced patient-physician contact, demographic transitions leading to questioning of traditions and authorities, population increases affecting the doctor-patient ratio and thus reducing the allotted time for each patient, and finally, commercialization of medical care reducing accessibility due to cost and focus of doctors and corporate bodies on profit over care [4].

The understanding and increased importance of medical professionalism and its implementation within medical settings is not new. In the US, from the late 1960s, two major pathways of categorizing and understanding professionalism emerged. The first is explained by the bioethicists as mainly concerned with a person’s rights and principles. This approach to professionalism asks the questions: “What should I do, and how should it be done?” The main principles involve the concepts of autonomy, justice, beneficence and non-maleficence. Secondly, professionalism is understood to be a virtue-based practice with documented codes such as the Hippocratic Oath. In this approach, the individual asks the questions: “What kind of person should I be in order to fulfil my professional obligation?” [5]. Over the years, a number of review articles and studies have been published on the conceptualization of professionalism but the topic remains complex. Therefore, in the 90s, the American Board of Internal Medicine (ABIM) sought to organize the understanding of medical professionalism by combining the main tenets into a standardized list. This list of the core competencies of medical professionalism have since been validated in various countries across the globe [6]. However, the components that define professionalism should include not only these standardized components from ABIM and the philosophical approaches noted above but also crucial to our understanding is the cultural context from which the medical professionalism is conceived and utilized [5, 7–11]. Recently, professionalism has also embraced a concept called professional identity formation [12–14]. Professional identify formation is where an individual physician reflects on what the profession requires of him or her. This concept forms a continuum which follows the learning concepts explained by Argyris and Schon [15]. These learning concepts, introduced by behaviorists in the 60s, are categorized into the single-loop, the double-loop and finally, the triple-loop of learning. Whereas in single-loop, what was required was of the doctors was professionalism in their behaviour, the double-loop period began to conceptualize what it is and why it is needed, the triple-loop of learning concepts even delve deeper in a reflective way on what is presently referred to as professional identity formation.

To date, studies and reviews on professionalism have been mainly published from within Western, Eastern and Middle Eastern cultures. It has been previously noted by numerous authors that though most of the domains of professionalism converge across cultures [16], there are many domains that vary greatly across different cultures [5, 9–11, 17, 18]. The variations noted depended on the dominant societal philosophy and cultural norms. As well, previous studies of professionalism have focused on the views of one cadre of health care worker, for example only students or only consultant specialists. In order to inculcate a Kenyan surgical practice that upholds professionalism, we first need to understand the views of clinicians, trainees and patients in regards to professionalism within our context. Then we can avoid introducing professionalism concepts that do not apply to the local context and hence are not successfully taken up. Therefore, this study aimed at exploring the concepts of professionalism that are prevalent in the Surgical Teaching department at Kenyatta National Teaching and Referral Hospital in Nairobi, Kenya.

## Methods

### Setting and population

The study population was drawn from the faculty and students of the Department of Surgery at the University of Nairobi, and from the patients and staff of the surgical wards at Kenyatta National Hospital. The study cohort included consultants, registrars, medical students, nurses, physiotherapists, occupational therapists, nutritionists and patients.

The University of Nairobi is the oldest and largest professional health care training institution in Kenya. It offers training in a variety of health care disciplines to approximately1500 students annually, through 6 undergraduate and 18 postgraduate programs. Until 7 years ago, it was the only institution in Kenya that trained surgeons. As of 2015, the Department of Surgery had 12 professors, 4 senior lecturers, 17 lecturers and 7 tutorial fellows that are trained and are training in the following subspecialties: general surgery, otorhinolaryngology, neurosurgery, plastic surgery, cardiothoracic surgery and paediatric surgery. The main clinical training site is Kenyatta National Teaching and Referral Hospital. The department of surgery teaches undergraduates students in medicine (3rd and fifth  years) and dentistry (3rd years), a total of more than 400 students per year. The surgical post-graduate training is a five-year program with year one being exclusively basic sciences. The clinical years (years 2–5) have approximately 55 students per year. The model for the curriculum includes traditional lectures with apprenticeship training in clinical settings and surgical electives.

Ethical approval to conduct this study was obtained from the Kenyatta National Hospital-University of Nairobi Ethics and Research Committee (Reference Number P59/11/2013).

### Study design and sampling

This was a sequential mixed method cross-sectional study comprising both a qualitative and a quantitative element. The qualitative element of the study involved an in-depth interview and focus group discussions among the surgical teaching community of the Kenyatta National Hospital and hospital surgical patients and was conducted first. This method was used in order to capture the cultural values of the participants associated with the conceptualization of professionalism within the Kenyan health care context. A quantitative phase was then employed using a cross-sectional survey method for triangulation and confirmation of the findings within the same surgical environment.

Convenience sampling was used to enrol participants for both the focus group discussions and key informant interviews. The focus- group discussions included participants from among faculty, registrars, medical students, nurses, physiotherapists, occupational therapists and nutritionists. For the key informant interviews, the cohort was drawn from senior faculty as well as patients. Patients were included as participants in the interviews in order to obtain views from those receiving medical professional services as well as the delivery providers. The patients were chosen from a 45-bed surgical firm, one of the three surgical firms, at Kenyatta National Hospital.

The inclusion criteria for participants for the focus group discussions and key informant interviews included the following: 1) willingness to participate and 2) gave written consent. For faculty who volunteered to be interviewed the following criteria was also followed: 1) faculty who had been in the department for greater than 20 years. For patients, they had to meet the following criteria: 1) give written consent to participate and 2) were able to verbally communicate without difficulty. The written consent for both the interview and the survey was obtained.

Convenient sampling was also used for the survey arm of the study within the same study population. Any individual who gave their written consent was given a questionnaire to complete. There was only 11 out of 204 (5.3 %) of the interview participants who also participated in the survey phase of the study.

### Data collection and analysis

Grounded theory method of data collection and analysis was used for the qualitative arms: the focus group discussions and interviews. The guiding question for clinicians was “In your view, what are the main themes or main issues that come to your mind when the word professionalism is mentioned in relation to your surgical work?” The main question for the patients was “How would you describe a professional doctor?” The interviewee was given a chance to share their view on how professionalism has been taught, allowed time to give any clarification and prompting by the researcher was given when needed. Follow up questions given by the researcher were used to clarify the views and the concepts from both focus group discussions and in-depth interviews as needed. There were 10–12 participants in each focus group and each person in the group was also given the chance to give their view before discussion and clarifications on the various responses was encouraged by the group as a whole. The data was collected by tape recording using Sony ICD BX112 2GB during the interviews. The Principal Investigator facilitated all the focus group discussions while a research assistant recorded the events and kept written notes. The taped recordings were transcribed into coherent English statements which were then entered into Atlas 5.2.0 by a research assistant to allow the development of the codes. The codes developed after the first interview were refined in the subsequent interviews and new ideas extracted until it was felt that the codes were mentioned in a repetitive fashion and no new ideas were being obtained (this is referred to as saturation).

After saturation occurred, codes were categorized into themes. These codes were then used to develop questions for the questionnaire that was used in the survey questionnaire. The questionnaire was initially piloted among five faculty members. It was then further revised into a 20 items that was distributed to 250 participants. The question posed for the participants was “What are the behaviours that best describe professionalism in regard to surgical practice and teaching?” The participants were given a 4-point Likert scale: strongly agree (4), agree (3), disagree (2), strongly disagree (1) to respond (see Appendix).

Descriptive statistics were used to analyse responses from the survey to identify measures of central tendency for each main item along with standard deviation. Factor analysis with promax rotation and Kaiser-Meyer-Oklin Normalization was used to explore the structure underlying the 20 items in the survey. Kaiser criterion was used for dropping the least important factors from the analysis when Eigen values were less than 1.0. Internal reliability was determined by employing Cronbach’s α. Kerandall’s tau b was used for correlation with independent factors such age, cadre, gender and number of years in the institution. The items were then matched qualitatively to the ABIM domains of medial professionalism [6].

## Results

### Domains generated from the FGDs and interviews

There were 16 focus group discussions: 9 groups with undergraduate students for a total of 104 participants; 2 groups with faculty for a total of 24 participants; 2 groups with nurses, nutritionist, physiotherapist and occupational therapist for a total of 22 participants, 3 groups with surgical registrars for a total of 36 participants giving an overall total of 186 participants for the Focus Group Discussions.

There were 18 key informant interviews: 10 interviews with senior faculty and 8 interviews with individual patients. The in-depth interviews generated about 23 concepts that the participants reported as reflecting the main component of professionalism. They were as follows, in descending order of frequency of reporting: Respect between colleagues and for patients (reported 71 times), proper and appropriate communication between colleagues and with patients (reported 62 times), the attitude/character/morals of the physician (reported 48 times), ethical values (reported 46 times), knowledge level and skilfulness of the doctor (reported 38 times), honesty (reported 31 times), integrity (reported 23 times), empathy (reported 21 times), quality of care given to patients (reported 14 times), confidentiality (reported 15 times), time keeping (reported 14 times), social skills in interpersonal interaction (reported 8 times) availability and etiquette with good grooming (both reported 7 times), commitment to work ethic (reported 6 times), consulting other peers/colleagues where and when necessary, putting patient first and following correct and ethical boundaries (all reported 5 times), do no harm, and equality (both reported 4 times), accountability (reported 3 times) and finally, justice and humility (both reported 2 times).

Using discourse analysis, the 23 different responses were then coded into three main themes: 1) *character in practice* (which comprised respect, empathy, keeping societal values and treating people with equality), 2) *concern for the patient* (which comprised upholding patient rights, justice, loyalty to the patient and having good knowledge of your field) and 3) *excellence in service* (which included commitment to ones job as a doctor, quality of care, honesty toward patients, integrity, responsibility, accountability, time keeping and better communication). For clarity, we have included below examples of responses that were categorized into each of the three themes to further delineate and clarify the themes:

1. *Character in Practise*
  - *Respect*
    One of the main concepts in this theme was respect. The participants’ placed emphasis on the relationships between patients and doctors and in particular, the values and virtues of the doctor that would maintain that relationship. The responses involving respect categorized respect as between the doctor and his/her patient and/or between colleagues. Respect is an important cultural value within Kenya.
    *“We are trained as surgeons to respect patient, from the time you meet the patient, there is need for us to give the patient the utmost respect…we should be the servant of the patient. “Key informant faculty −1 (KIF-1).*
    *“I think that respect should not only be junior-senior but between different cadres, for example doctor nurse and even doctor-physiotherapists, doctor –cleaner, because that is something we are seeing in other facilities most of there is always that respect which I can say in our facility it is not totally up there”* FGD Registrars Group 1(FGD R-1)
    *“In the African culture one is expected to respects his seniors”* FGD R-2
    *“Consideration for fellow colleague that is a cultural thing that should be taught in the house, unfortunately there the home are breaking down” FGD Faculty Group 2(FGD F-2)*
    *“Respect to seniors has always been part of what we practiced while young by giving away seats, being corrected by older neighbours because. In school this is seen in students respecting their teachers as well as older nurses and older patient in the ward” (KIF-2)*
    It was often commented by participants that this concept, though they were reporting it as important, was often not practiced by those who they saw as role models. This is seen in the following examples of negative responses.
    *“Professionalism will require certain level of respect, I agree that I may be not as learned as you but I am still a man, I am a human being. There is a certain level of respect that you should accord me.” FGD Auxiliary Group 1(FGD A-1)*
    *“Respect one another in that you are not rude to the other doctor and if a doctor, for example, has made a mistake, publicly, I feel it’s nice that you call that doctor aside, you talk to them, as opposed to embarrassing someone”* FGD R-3
    *“The senior should not yell and shout and embarrass fellow colleagues in front of patients, I think part of being professionalism comprises respect" FGD Medical student group 1(FGD M-1)*
2. Concern for patient
  The participants also responded that professionalism should include a concern or empathy for the patient. This was expressed in terms of patient rights, skills to care for the patient in the particular area of speciality and justice towards patients. It was repeatedly mentioned by participants (46 times) that ethical behaviour in medical practise is also a key concept of professionalism. Further, the participants repeatedly stressed a need to follow guidelines and regulations from the Kenyan Medical Practitioners and Dentist Board that outlines an ethical code of conduct for doctors. Examples of responses that follow this theme are given here:
  *“Professionalism to me is understanding and applying the principles of ethics in your conduct with your colleagues and patient.” FGD R-1*
  *“Thinking about adhering to the rules and regulations of the institution.” FGD Auxiliary Group 2(FGD A-2)*
  *“Professionalism is like values and standards that somebody who is in the medical field is expected to have and abide by whenever he is dealing with people.”* FGD F-2
  When the researchers inquired in regards to resources available to the participants on the topic of medical ethics and professionalism, they were well versed in guideline sources such as those available from the Kenyan Medical Practitioners and Dentist Board.
  *“*The *board has a book on professional conduct, and it outlines, how a medical professional doctor should behave.”* Key informant interview with board representative*.*
  The patients, students, nurses and surgical teachers/faculty were in agreement that a physician should have good clinical skills and knowledge. Many participants reported this as an essential component of professionalism for a surgical career (38 times).
  *“In theatre, you have to do the best possible, and that’s why you have to have a training which really prepares you for that. When you are in theatre you are practically alone with your patient who can answer your questions. You can’t ask the patient, “Can I cut here? Shall I remove this or that or the other one,”* KIF-2
  *“A good doctor is one who is able to diagnose my problem and treat me well”* Key informant Interview with patient 1(KIP-1)
  *“But I think for me first and foremost you must be a qualified personnel for that particular field of profession, in this case you must be qualified in a recognized university”* FGD A-2
3. Excellence in service
  The theme of excellence in service, includes the concept of concern but as it is used here, it refers mainly to the quality of service given together with a humanistic handling of the patient. The “bedside manner”, as it has been referred to, should include an approach of open communication with the patient. Doctor-patient and doctor-doctor interactions were recorded as requiring respect, but the study participants also recorded communication (62 times) as a major component of professionalism. Correspondingly, the informed consent was frequently reported by participants as a surgical task that should be performed well despite the fact that many participants reported it was often performed poorly, if at all. It was noted by participants that the consent should include both explaining to the patient the nature of their illness, as well as a respectful manner in the way in which it is explained.
  *“If I am taking a patient to theatre I will have to talk to the patient about their condition, make them understand it, find out if they have understood it, explain to them about the complications in a way that they understand in an empathetic way.”* FGD R-2
  Respondents also noted that an improperly performed consent process was a lost opportunity for role modelling appropriate professionalism. This comment was reported repeatedly by faculty (6), nurses (4), students (3) as well as patients (2).
  *“The patient should give you informed consent. Well, the reason why it is informed is because you should inform the patient what you want to do, but most of us just say, well can you sign here? Are we allowed to do the operation, yes, just sign here, without explaining to the patient what are the consequences of that and so on”?* KIF-2
  *“A patient who had a terminal disease was told by another doctor “you are not going to live; you will die after ten days.” Then after ten days the patient was still alive, from the patient’s side, the statement from the doctor seems harsh and uncaring, the way of explaining is the difference, there are people who can tell the patients in an indirect way in which they can be satisfied “FGD R-2*
  *“I do not know the nature of my illness till now, they mentioned a word during a round check but not to me.”* KIP-2
  The following are examples of the other concepts less commonly reported by study participants: honesty, morality, integrity and empathy.
  *“I think I’d like to put it this way-- I’d like to talk about three things: first of all, the attitude, the moral behaviour and the context though when you’re talking about the context”* KIF-4
  The mode of service delivery and whether it included integrity, honesty, compassion, empathy and responsibility was also reported as a component of professionalism.
  *“I think for me professionalism is about delivering quality care, that you put the patient first, and in order for you to do that, you must have integrity, because there is no way you are going to do this without integrity, you cannot separate the two issues, so if you put your patient first and that you want to provide the best possible care for that patient then you have to do it within the ethics that govern the profession”* FGD F-2
  *“Well, for me it’s about honesty because for a surgical patient usually most of them they are like at your mercy so you need to be honest with them in terms of whether you are capable of handling whatever illness it is that they have and also in telling them what exactly the problem is or whatever the illness is so for me it’s about honesty.”* KIF-5
  *“Mine may come from different level of thought, maybe from what we are saying that we should be empathetic, kind, gentle, humble, all those good traits.”* FGD R-3
  Professionalism was also noted to include accountability, time keeping, dedication, commitment, and availability by study participants.
  *“Accountability and ensuring that you carry out your duties in a timely manner. I look at it in terms of commitment to deliver, and accountability to the people who are receiving your services as well as accountability to the people in the profession”* FGD F-2
  *“We call common courtesy that you extend to a human being in general, so, for example, time keeping, keeping your word”* KIF-6
  Though it appeared by the responses recorded that many participants understood fairly well the various components of professionalism, the responses reflected a large gap between that understanding and actual surgical practice. This could be referred to as a form of hidden curriculum or a parallel narrative, that forms a divide between what the participants think/understand/believe and the acts of professionalism taught by clinical example.
  *“Because sometimes doctors are missing in action, probably because they are busy chasing greener pastures elsewhere so I think sometimes doctors are not disciplined and that is not professional or they offer to do procedures and they know that whatever they are doing does not even add the quality of the patient’s life but they just do it because they think there is money to be minted. So I think a good doctor is disciplined”* FGD M-2
  *“Reliability is that I can come to you on any time or any occasion and expect a certain level of care and I won’t expect next time you are moody; you won’t be talking or the other time you won’t even…”* FGD A-2
  *“Dedication to your work, you don’t want to say you are a doctor practicing medicine when half the time you want to be concentrating on other things. Maybe you want to do your farming in the village, if you want to be a doctor, just be a doctor. That is professionalism”* FGD F-2
  Many study participants reported dedication because of the demand that surgery often requires. Similar to this concept, it was also reported that professionalism should include the concept that surgery as a vocation is more of a “call” than strictly a profession.
  *“*W*e have been trying to run away from the fact that the medical profession is slightly different from the other professions, because in the other professions I come to work at 8 am and leave at 5 pm but in the medical profession there is that additional aspect that people say it's supposed to be a calling where you go an extra mile other than just doing your routine work*…*”* FGD R-3
  *“Should keep the patients first before anything else, avoid things like the striking and all that”* FGD A-1
  Some study participants mentioned grooming as part of professionalism.
  *“Professionalism should also entail the doctor’s etiquette, his mode of dressing and his attentiveness. Is he paying attention to the patient, is he using his phone when the patient is talking to.”* KIF-9

### Survey results

**Fig. 1:**
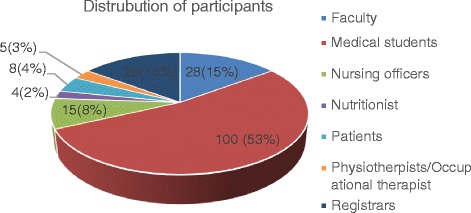
The frequency distribution of the participants by health care worker or patient groups

Of the 250 questionnaires sent out, 188 were completed (75 % response rate). Majority (53 %) of the respondent were medical students (Fig. 1). The mean age of the survey participants was 31.24 +/−9.8 years with a male–female ratio of 1.7:1.

The frequency of the responses to the 20 attributes of professionalism on the survey is summarized in Table 1. The two items most frequently chosen as important were respect and having good clinical knowledge and skills in your chosen field. Empathy and dedication to your career were reported as the least important items in professionalism. A large number of attributes (17/20) were given a rating of “strongly agree” by a majority (over 50 %) of survey respondents. But a number of the professionalism attributes, previously chosen through the FGDs and interviews, were not rated highly as attributes of professionalism by survey participants. 0.5–2.1 % of respondents reported “strongly disagree” on 11 of the 20 items. For the total 20 attributes listed, between 1.1 and 10.6 % of respondents reported “disagree” with the importance of that attribute toward professionalism in a Kenyan context. Hence, there is still a wide range of knowledge and opinions on what is medical professionalism and how it is presently and how it should be implemented in the Kenyan medical setting.

**Table 1:** Frequency and means of response on various items during the survey

*n* = 188	Strongly agree (%)	Agree (%)	Disagree (%)	Strongly disagree (%)	Mean
Respect	74.5	22.3	2.7	0.5	3.71
Having good knowledge and skills in that field	71.3	27.7		0.5	3.71
Do no harm	69.7	29.3	1.1		3.69
Keeping the standard and quality of care	69.1	26.6	4.3		3.65
Upholding the right of the patient	68.6	27.1	3.7		3.65
Integrity	66.5	30.3	2.3		3.65
Commitment	66	27.7	3.2	1.6	3.61
Treating the patient with equality	64.4	30.9	2.7		3.63
Communication	63.3	29.8	4.3	1.1	3.58
Accountability	60.1	32.4	4.3	1.1	3.55
Etiquette	57.4	37.2	2.7	0.5	3.55
Keeping time	54.3	30.3	10.6	2.1	3.4
Justice	54.3	39.9	3.7		3.52
Keeping time	54.3	30.3	10.6	2.1	3.4
Availability	52.1	38.8	7.4	1.6	3.4
Humility	52.1	38.3	8.5		3.44
Keeping societal values	50.5	43.6	4.3		3.47
Honesty	49.5	42	5.3	1.1	3.43
Empathy	41.5	51.6	5.3		3.37
Dedication	41	47.3	10.1	0.5	3.3

Kaiser-Meyer-Oklin Bartlett, the measure of sampling adequacy for factor analysis, was 0.924 (*p* = 0.000), meaning the sample is statistically adequate to perform a factor analysis. The reliability, consistency of how closely the items are related, among the items showed a Cronbach’s α of 0.927, implying excellent internal consistency of the various items. Three factors had Eigen values greater than 1, meaning there were 3 main underlying factors that can account for the total 20 items.

Factor analysis was used to explore the 20 items to get the underlying factors. Three (3) factors accounted for 61.2 % of the variance. The loading of items indicated the following factor structure (Table 2). The first factor, consisted of communication, responsiveness, and having character when serving others fitting into the theme of excellence in service. The second factor comprised justice, patient rights and doing no harm to the patient reflect a theme of patient-centred care. Lastly, the third factor included respect, keeping societal values, empathy and treating patient equally reflecting morality in practice as the underlying theme. The theme of the third factor is the one factor most closely in agreement with the respect domain in ABIM while that of the second factor is closely tied to the concepts of excellence, accountability and honor. The first factor has a mixture of duty, honor and contains practice etiquette which is not an integral part of the ABIM domains of professionalism.

**Table 2:** Factor loading, Eigen values and percentage variance of the 3 factors loaded by twenty items of professionalism matched to ABIM (American Board of internal Medicine) domains

	Excellence in service	Patient oriented	Morals in practice	ABIM
Empathy			0.618	Respect
Respect			0.484	Respect
Societal values			0.848	Respect
Equality			0.402	Honor
Having good knowledge and skills in that field		0.923		Excellence
Justice		0.532		Honor and integrity
Being loyal to patient/do no harm to the patient		0.693		Accountability
Upholding the right of the patient and of the doctor		0.597		Honor and integrity
Communication	0.579			Excellence
Keeping the standard and quality of care	0.507			Duty
Commitment to your work as a doctor	0.654			Duty
Accountable to others	0.705			Accountability
Dedication to patient/putting patient first above one’s need’s	0.646			Altruism
Integrity and responsibility	0.799			Honor and integrity
Being honest to client	0.762			Honor and integrity
Humility	0.661			Accountability
Etiquette	0.511			Practice habit
Keeping time as a doctor	0.791			Excellence
Eigen Values-Total	9.4	1.3	1.02	
% of Variance	49.26	6.60	5.36	
Cumulative	49.2	55.86	61.23	

In the survey, there was only one statistically significant association between gender and the likelihood to report a particular concept of professionalism: females more frequently reported the upholding the right of the patient and the rights of the doctor as compared to males (*p* = 0.002, χ^2^ test).

An increasing age of the participant was correlated with an increased likelihood to report one of the following items: treating patients equally, respect for colleagues and patients, integrity, and responsibility with Kendall’s tau-b of 0.146, 0.195 and 0.183 and a *p*-value of 0.022, 0.001 and 0.003 respectively. Chi-square also showed a statistically significant associated between age and reporting the item, upholding the right of patient and doctors (*p* = 0.035).

The number of years a health care worker participant had been employed in the institution had a statistically significant correlation with the following domains: treating patients equally, respect for colleagues and patients, being accountable to other colleagues and integrity and responsibility (Kendall’s tau-b of 0.156, 0.165, 0.152 and 0.120 respectively with *p*-value of 0.010, 0.003, 0.010 and 0.049 respectively). There was also statistical significance with regard to the number of years the participants had been in the institution and having good knowledge and skills as a concept of professionalism (χ^2^ test, *p* = 0.035,). There was a linear relationship between age and the responses noted above as the longer the participant had worked in the institution the more likely they were to mention these items.

Also, the cadre of the participant had a statistically significant correlation with the attribute of accountability, with more of the students, nurses, physiotherapists, occupational therapists and nutritionists mentioning accountability as compared to other cadres (Kendall’s tau-b of 0.126 (*p* = 0.045).Having good knowledge and skills, humility and etiquette as concepts of professionalism were reported significantly more often by faculty than by other health care workers (χ^2^ test *p*-value of 0.026, 0.026 and 0.011 respectively). There were no other statistically significant correlations found between the cadre of the participant and other attributes in the survey.

## Discussion

The findings in this study suggest that many of the domains of medical professionalism found in the ABIM were also found in our study in the Kenyan context: excellence in the care of the patient, concern for the patient and character in practise. However, respect as a component of professionalism, repeatedly had the highest frequency of responses by participants throughout the study. The survey indicates that being female and increasing age was associated with mentioning respect and justice factors.

One interviewee is quoted as saying “*it is African to respect*”. The depth of respect within Kenyan society can be illustrated by the use of a special word that is solely for greeting seniors and which cannot be used for peers or those younger. This greeting, “*shikamoo*”, is used throughout coastal Kenya, in particular. Respect was depicted as being that of acquiescent towards the seniors, in particular, students toward faculty. The practice of respecting seniors is based on the belief system that they are the custodians of wisdom and advice. But more than that, Africans believe in the connectedness of the world between the living and the dead which can determine ones blessings or curses in life [19, 20]. The implication is that those who are older, are able to place a blessing or a curse on the younger person, depending on the degree of respect they do or don’t receive from them.

Respect between a doctor and his/her patient can be demonstrated through caring. Caring is recognized by being available, empathetic and being able to explain to patients their condition in a manner they can understand. Further, the patients are treated with respect as people because they are considered to be nearer to death, and the dead play a major role in the life of the living within African culture [19]. African culture is known for its care of the vulnerable [21, 22]. However, in the commercialized world, patients are often seen as customers hence some doctors get busy in private practise [1].

In their review of the worldwide cultural constructs based on Hofstede’s work [23], Wursten and Fadrhonc [24] place Africa in the pyramid cluster. The cultural constructs in this cluster include high power distance index among other indices. This implies that, in Africa, people who are less powerful accept that power is distributed unequally and are subservient to the powerful, and hence are more likely to give them respect based solely on their position. This is consistent with our results where respect was a highly important attribute in comparison to other attributes that were listed as essential for professionalism.

The conceptualization of professionalism as being excellence in service as is seen in this study implies a desire for positive clinical outcomes. This outcome could take the form of quality of care and monitoring of outcomes for purposes of improvement and learning. The developing world rarely reports on their audits [25, 26], hence this would imply influence from medicine as practised in the Western cultures. Historically and up to now, the main surgical teachers and those who continue to write most of the surgical books that the Kenyan surgeons will read come from a western culture perspective. That influence should not be underestimated, as levels of literacy increase.

However, given that professionalism is a cultural construct and a social contract between doctors and the society [27], the philosophical undertones of a culture will certainly colour the themes. This study showed that professionalism in the Kenyan culture emphasizes respect. Though all the other domains were mentioned, respect was mentioned higher throughout the study, both in the interview and the survey.

In the literature, the impact of the health care workers culture of origin on professionalism has been studied in numerous cultures. A survey from the Arabian world (Egypt, Saudi Arabia and United Arab Emirates), among 45 health care professionals, revealed autonomy of the professional as important. This reflects the paternalistic dominance seen in the Middle Eastern cultures. In Taiwan, using the nominal group technique of interview among 91 participants, the Confucian cultural framework of integrity/honour of the individual found to be strongly emphasized by the study participants when asked about professionalism. Cohen, an American author, in his review on the definitions and roles of medical schools to ensure the next generation of doctors are professionals, emphasizes the use of the ABIM document for its definition of professionalism. This document is based on Western cultural norms where the concept of the individual and their rights take precedence. Hence, one of the main principles found in the ABIM document is patient autonomy, a reflection of the Western Hippocratic tradition within the American medical culture.

The main themes that we found in this study revolve around character in practice, concern for the patient and excellence in service. Though various individual components of the “excellence in service” factors were only mentioned a fewtimes in the interview or were less often reported as “strongly agreed” in the survey, the overall high number of participants who did report at least “agreeing” or were “neutral” statistically gave this theme a stronger factor variance than the other themes. These components are reflective of the humanistic domains of professionalism. Despite the more moderate reporting, it still reflects many similarities between our study and the domains found in the ABIM document. Tsugawa et al. [16] notes in their study that there is a lot of similarities across cultures on how they perceive professionalism and is likely the ability of the ABIM components to have been validated in many countries. However, in our study, the lower reporting overall on many ABIM domains indicates a preference of Kenyan practitioners to perceive respect as a major domain as opposed to the humanistic domains.

Another area where the ABIM list of professional attributes and our study is similar is in the theme of excellence of care, in particular in clinical skills and knowledge. Professionalism as was defined by Pellegrino [1], has three elements; acquisition of skills and knowledge, self-regulation and a shared commitment. In our study, participants reported frequently that for one to be a good doctor, they should be able to able to perform their work skilfully. This mirrors themes such as excellence in duty as found in the ABIM framework and has been suggested by authors like Chandratilake [18].

Confidentiality was mentioned rarely (only 12 times) in the interview. This is again likely a reflection of Kenyan culture where the African *Ubuntu* ideology of “I am because we are” makes it difficult, in most setting to value confidentiality. Ubuntu is seen when the relatives of the patient and the extended family demand to be present at all times and to be fully informed of the patient’s condition, irrespective of the patient’s desire and many times, without his or her consent. This same lack of importance of patient confidentiality was also noted in an earlier article by Baigana et al. in Uganda [28].

Many participants reported an understanding of professionalism but also noted their main experience was actually a paucity of these attributes. Further, many participants reported the presence of negative attributes such as differential attitudes and behaviours by some doctors towards private patients as compared to non-private patients. This reported paucity of positive professional attributes and the presence of negative attributes has been previously noted by other authors in low to middle income countries. Corruption both in terms of accepting gifts as well as taking time meant for patients in public care was noted in Estonia and South Africa [29, 30]. This is in keeping with our findings that the attribute of altruism was not mentioned even once by any of the study participants.

Professionalism, even in the form of the most important attribute reported, respect, was seen as lacking by the study participants. For example, respect was reported as not being practiced by senior health care workers towards their junior counterparts. This caused frustration by the junior workers who felt that they had no mechanisms by which to be able to change the situation. The study results portray an environment where juniors do not question their seniors. Similar environments have been reported in Japan and in Turkey, where the hierarchical cultural/social systems allow unethical behaviours by seniors to go unreported and unquestioned by the juniors. This propagates an environment of ongoing negative professional attributes because though what should be done is known, the juniors see conflicting behaviours and then model it when they become seniors [31, 32].

These factors agree with the definition given by Epstein and Hubert, that “professional competence is the habitual and judicious use of communication, knowledge, technical skills, clinical reasoning, emotions, values, and reflection in daily practice for the benefit of the individual and community being served” [33]. This definition would fit well into the Kenyan culture with the addition of respect. In this study, the decreased emphasis on certain components of the ABIM model demonstrate the impact of different cultural concepts. It also solidifies the need for concerted efforts to teach professionalism with incorporation of the tenets of the specific cultural context despite the delivery of many of these traits generically across geographic regions.

One limitation of our study design is an overlap between interview participants and survey participants. Participants who were part of both phases of the study could have entered bias into their survey answers because of the influence of the earlier interview. However, the number of participants, as noted earlier, was very small and the duration between the two phases was significant being not less than six months. A second limitation of our study was the non-random accrual of study participants. We may have an overrepresentation of people who had either strongly negative or positive thoughts about professionalism as this may have been their impetus to agree to participate. This study may not account for views of individuals, workers or patients, who know little or nothing about professionalism as it is possible that they were more likely to not volunteer for a study about a topic they don’t know or understand. Therefore, future research could include an investigation of the magnitude of awareness or lack thereof of these professionalism concepts on a larger population-based or random sampling basis.

## Conclusion

This study reveals the perceptions of surgical professionalism, as held by health care workers and patients, within a Kenyan cultural context. The major pillars are respectful service provided by skilful clinicians. This study shows that teaching internationally recognized components of professionalism to overcome gaps is crucial but that attribute uptake will be more successful if the local cultural context is also well understood and incorporated.

## Appendix

**Table 3:** The following behaviour best describes professionalism in so far as surgical practice and teaching is concerned?

Item	Strongly agree	Agree	Disagree	Strongly disagree
Empathy				
Respect of patient and colleagues				
Keeping the societal and community values				
Having good knowledge and skills in that field				
Justice				
Being loyal to patient; do no harm				
Upholding the right of the patient and of the doctor				
Treating the patients the same without discrimination/equality				
Good interaction between doctor and patient and doctor –doctor/health care workers				
Keeping the standard and quality of care				
Commitment to your work as a doctor				
Accountable to patient and other doctors				
Dedication to patient/putting patient first above ones needs				
Integrity and responsibility				
Being Honest to client/owning to ones mistakes				
Humility				
Etiquette				
Keeping time as a doctor				
Availability for patients and students				

## Abbreviations

FGDs: Focus Group Discussions
ABIM: American Board of Internal Medicine
FGD M1-9: Focus Group Discussion with medical student’s groups 1–9
FGD F1-2: Focus Group Discussions with faculty groups 1–2
FGDA1-2: Focus Group Discussions with nurses nutritionists, physiotherapists, occupational therapists’ groups 1–2
FGD R1-3: Focus group discussion with residents group 1–3
KIF 1-10: Key informant interview with senior faculty 1–10
KIP1-8: Key informant interview with patient 1–8
